# Supplementary material for: Comparison of longitudinal PSQI and actigraphy-assessed sleep from pregnancy to postpartum
Source: PLoS One. 2025 Jun 18;20(6):e0323489. doi: 10.1371/journal.pone.0323489 (PMC12176231; doi:10.1371/journal.pone.0323489)
Supplement: Attachment — Submitted filename: comments.docx [file pone.0323489.s002.docx]

Abstract

1. The abstract has no introduction
2. The statistical methods used to analyze differences between actigraphy and PSQI measures were not explained.
3. The cause of this difference was not explained
4. The recommendation is not in line with the objective

Introduction

1. Line 65, 66 , reference 2, 4 is an old reference
2. The paragraph “Six studies in the United States have examined changes in parental sleep from pregnancy 93 to postpartum using actigraphy [24-29], of which four had small sample sizes of ≤30 participants 94 [26-29]. Additionally, none of these studies have examined sleep after 16 weeks postpartum, 95 when many sleep complaints do not begin to occur until the third trimester (after 28 weeks) and 96 few report sleep metrics beyond total sleep time and sleep efficiency.” is not necessary. It is better to for the introduction focus on the main objective.
3. The result of previous studies was not present quantitatively, and need explanation on the type of difference.
4. The limitation of measurement error from actigraphy and their cause was not explained as a comparison with the gold standard or patient report.

**Method**

Well written

Appropriate statistical analysis was done, with better interpretation.

However, the explanation related to PSQI (Line 141-149) is too broad, write only the main ideas
